# Comparing the Effects of Dietary Flaxseed and Omega-3 Fatty Acids Supplement on Cyclical Mastalgia in Iranian Women: A Randomized Clinical Trial

**DOI:** 10.1155/2014/174532

**Published:** 2014-08-13

**Authors:** Farideh Vaziri, Mansooreh Zamani Lari, Alamtaj Samsami Dehaghani, Mousa Salehi, Hossein Sadeghpour, Marzieh Akbarzadeh, Najaf Zare

**Affiliations:** ^1^Community Based Psychiatric Care Research Center, Department of Midwifery, Fatemeh (P.B.U.H) School of Nursing and Midwifery, Shiraz University of Medical Sciences, P.O. Box 71345-1359, Shiraz 71936 13119, Iran; ^2^Student Research Committee, Fatemeh (P.B.U.H) School of Nursing and Midwifery, Shiraz University of Medical Sciences, P.O. Box 71345-1359, Shiraz 71936 13119, Iran; ^3^Infertility Research Center, School of Medicine, Shiraz University of Medical Sciences, P.O. Box 71345-1359, Shiraz 71936 13119, Iran; ^4^School of Nutrition and Food Sciences, Shiraz University of Medical Sciences, P.O. Box 71345-1359, Shiraz 71936 13119, Iran; ^5^Faculty of Pharmacy, Shiraz University of Medical Sciences, P.O. Box 71345-1359, Shiraz 71936 13119, Iran; ^6^Department of Biostatistics, School of Medicine, Infertility Research Center, Shiraz University of Medical Sciences, P.O. Box 71345-1359, Shiraz 71936 13119, Iran

## Abstract

Considering the negative side effects of chemical drugs, there is a great need for effective alternative treatment strategies to manage cyclical mastalgia. Therefore, this study aimed at comparing the effects of flaxseed diet and omega-3 fatty acids supplement on treatment of cyclical mastalgia. In this study, 61, 60, and 60 women, respectively, received flaxseed as bread, omega-3 fatty acids as pearl, and wheat bread as their diet for two menstrual cycles. At the baseline cycle and end of both interventional cycles, intensity of mastalgia was measured using visual analogue scale. Analysis of covariance showed a significant difference among the three groups regarding the mean intensity of cyclical mastalgia in the first and second cycles of the interventions (*P* < 0.001). Also, repeated measures analysis of covariance with adjustment of two variables of age and mastalgia intensity of the baseline cycle demonstrated that flaxseed bread was more effective compared to omega-3 and wheat bread (*P* < 0.001). The results of this study demonstrated that flaxseed bread diet was an effective approach in decreasing cyclical mastalgia and could be prescribed to women as a simple treatment with few complications.

## 1. Introduction

The most prevalent breast disorder which makes women consult with an informed individual, like a midwife, is breast pain or mastalgia. Mastalgia is in fact the reason for 30–47% of the referrals for clinical breast examination [1, 2]. In 10–30% of the cases, mastalgia is severe and causes disturbances in normal life; thus, it requires frequent evaluation and treatment [3]. It also leads to disorders in sexual, physical, and social activities in 48%, 37%, and 12% of the cases, respectively [1].

Breast pain may be cyclical, noncyclical, and extramammary (nonbreast). Cyclical breast pain is related to menstrual cycles and starts in luteal phase. The symptoms of cyclical breast pain include breast congestion, soreness, and feeling of heaviness and tenderness [4, 5]. Cyclical mastalgia might last for more than 7 days in 11% of women [2].

The etiology of cyclical mastalgia is not known; however, since it starts in luteal phase, hormonal stimulation might be the cause. High level of estrogen, low level of progesterone, and imbalance in estrogen to progesterone ratio are assumed among its causes, as well [3, 4, 6]. Bromocriptine and tamoxifen are considered as two effective drugs in treatment of cyclical mastalgia. Yet, bromocriptine is accompanied by some side effects, such as nausea, vomiting, and dizziness and the common side effects of Tamoxifen are hot flashes and vaginal dryness [4, 7]. These side effects have reduced the popularity of these drugs for treatment of cyclical mastalgia.

Therefore, similar to many other health issues, treatment of cyclical mastalgia has greatly moved toward herbal treatment and complementary medicine. Studies have indicated that using chasteberry is effective in treatment of cyclical mastalgia. However, using this herb has its own side effects [8]. The results of a large study by Goyal and Mansel showed that consumption of evening primrose oil with or without antioxidants was not significantly different from taking the placebo. Moreover, Britain has revoked the certificate of Efamast drug, which consists of gamolenic acid isolated from evening primrose, in treatment of cyclical mastalgia [9].

Flaxseed, scientifically known as* Linum usitatissimum*, contains essential unsaturated fatty acids, such as alpha linolenic acid, which are biological precursors for producing omega-3 fatty acids. Furthermore, flaxseed contains a large amount of plant lignans, a major class of phytooestrogens. Lignans have agonist/antagonist properties of estrogen receptors as well as antioxidant effects [10, 11].

Regarding the estrogenic properties of flaxseed, the study performed by Lewis et al. showed reduction in the intensity of hot flashes in the menopausal women who were using flaxseed [12]. Nonetheless, two recent studies have not stated the superiority of flaxseed to the placebo in decreasing hot flashes [13, 14].

C-reactive protein (CRP) is produced by the liver. The level of CRP rises when there is inflammation throughout the body. A recent research suggests that patients with elevated basal levels of CRP are at an increased risk of diabetes, hypertension, and cardiovascular disease [15]. Desirable effects of flaxseed or its derived lignans have been demonstrated on CRP level and blood lipids [16, 17].

The lignans existing in flaxseed inhibit aromatase enzyme activity with subsequent reduction in estrogen synthesis that are thought to contribute to prevention of estrogen-dependent cancers such as breast cancer. Another way by which lignans can affect sex hormones is binding to testosterone leading to its fast excretion [18].

The investigation carried out by Sturgeon et al. showed that flaxseed diet had no significant effect on reduction of the serum levels of sexual hormones, including esteriol, estrogen, and testosterone, in menopausal women [19]. In another study by Wang et al., 10% flaxseed diet in rats which had a graft of human breast cancer showed reduction in tumor growth and metastasis [20].

According to Goss, as cited in the review study by Rosolewich, flaxseed is listed as the first-line treatment of cyclical mastalgia [21]. However, the researchers of the current study could find no other clinical trials on the effect of flaxseed on cyclical mastalgia.

Furthermore, previous studies have indicated contradictory results in terms of the effect of omega-3 fatty acids on cyclical mastalgia. In one study conducted in Iran, omega-3 fatty acid decreased breast tenderness [22, 23].

Since flaxseed is an inexpensive, tolerable, and accessible seed [11] and its effect on cyclical mastalgia in the literature has been more anecdotal, the present study aims to investigate its effect on cyclical mastalgia and compare the results with those of omega-3 fatty acids.

## 2. Materials and Methods

### 2.1. Research Conditions and Participants

This clinical trial was conducted on 181 Iranian women within the age range of 20–45 years who suffered from cyclical mastalgia and had referred to health centers and gynecology clinics of Shiraz University of Medical Sciences, Shiraz, Iran. The health care providers practicing in these centers were asked to introduce the women suffering from cyclical mastalgia to one member of the research team who was responsible for data collection.

Data collection was started from February, 2012, and continued until September, 2013. This study was confirmed by the Ethics Committee of Shiraz University of Medical Sciences (code: CT91-6427). This investigation was also registered in the Iranian Registry of Clinical Trials as IRCT2013010510327N3.

The inclusion criteria of this study were as follows: willingness to participate in the study, mastalgia intensity of 4 or above based on visual analogue scale, regular menstrual cycles between 24 and 35 days, body mass index between 19 and 29, not being willing to get pregnant in the next months, not being in the lactation period, not having the history of any kind of breast tumor, having no history of any kind of tumor and cancer in other organs including endometrium and ovaries, having no history of breast cancer among the first-degree relatives, not being on a specific diet, having no history of any systemic and psychological diseases, not having allergy to flaxseed, and not taking any drugs for mastalgia. All the participants signed the written informed consents. The exclusion criteria of the study were as follows: probability of pregnancy, starting to take any kind of drugs and analgesics during the study, experiencing unexpected events including marriage, loss of close relatives, and surgery, starting any kind of new lifestyle such as exercise or diet, having allergy, intolerance of supplements, and frequent disruption of eating bread or supplements for 5 or more days.

### 2.2. Designing and Collecting Data

The researcher met the introduced women in one of the health centers or gynecology clinics in their neighborhood. In this study, cyclical mastalgia was defined as the breast pain which occurred at least two weeks after the menstrual period and mitigated at the beginning of the next menstrual period. Blommers et al. also used this definition for diagnosing mastalgia [22]. Therefore, the following questions were asked to diagnose cyclical mastalgia.

(1) Is your breast pain related to your menstruation? (2) Does your breast pain subside after the beginning of the menstrual period? (3) How many days does your breast pain last?

After checking the inclusion criteria, if it was 2 or 3 days prior to the subjects' menstrual period, mastalgia intensity was determined by visual analogue scale. In case mastalgia intensity was 4 or above and its duration was 5 or more days, the subjects were enrolled into the study. In case a participant was at the beginning or in the middle of her menstrual cycle, her mastalgia intensity was determined in the following visit at the end of her menstrual cycle. Final days of the menstrual cycle were selected for evaluating cyclical mastalgia in order to make the measurement more reliable, encourage the women to participate in the project, and start the intervention shortly after the interview, that is, at the beginning of the next menstrual cycle. The qualified participants were randomly assigned to three intervention groups, namely, flaxseed bread, omega 3 fatty acids, and wheat bread, through block randomization. In all these three groups, the intervention started from the beginning of the next menstrual cycle (with respect to the baseline menstrual cycle) and lasted for about two cycles. Intervention by flaxseed bread: This group received 30 g flaxseed on a daily basis. In this way, 30 g of ground flaxseed was given to the participants in form of three slices of bread. Each slice contained a mixture of 30 g wheat flour, 10 g ground flaxseed, and some water. Indian brown flaxseed which is available in Iran market was used in this study.

We did not measure the composition of the flaxseed used in our study. Instead we obtained this information from literature. The 10 g ground flaxseed supplement provided approximately 50 kcal, 2.4 g of protein, 3.6 g of fat (50–60%*α*-linolenic acid), 2.4 g of carbohydrate, and 2.2 g of dietary fiber (including 1.2 g of soluble fiber) [24]. In contrast to flaxseed oil, flaxseed is the most concentrated food source of the plant lignan secoisolariciresinol diglucoside (SDG). In humans and other animals, ingested SDG is converted by bacteria in the colon to the biologically active lignans enterodiol (END) and enterolactone (ENL) [25]. Li et al. reported that the SDG content in dried flaxseeds was 15.4 mg/g. So we estimate that each participant in the present study took 462 mg SDG and also 6.38 g omega-3 fatty acids daily [26, 27].

The participants could eat the bread slices in one or three meals as they liked. The bread was prepared by Hadis Bread Company in Shiraz. The participants were provided with the bread slices on a weekly basis and they were asked to keep them in the refrigerator or freeze them until the consumption date. The researcher arranged the reception of the bread and reminded the participants of its daily consumption via telephone.

Intervention by omega-3 fatty acids: in this group, one pearl of omega-3 fatty acids, which contained 180 mg eicosapentaenoic acid and 120 mg docosahexenoic acid and was produced by Dana Pharmaceutical Company in Iran, was given to the participants every day. The pearls were given to the subjects based on the approximate consumption dosage in one cycle. Also, the participants were reminded of taking the supplement through telephone contacts.

Intervention by wheat bread: in this group, three slices of bread, each of which containing 40 g wheat flour, were given to the participants every day for two menstrual cycles. The same companies produced flaxseed and wheat bread. The intervention method was also the same as that of flaxseed. In both of the intervened menstrual cycles, the researcher visited the participants 2 or 3 days prior to the beginning of their menstrual periods. During the visit, the researcher evaluated and recorded the manner and amount of consumed bread or supplements and their side effects. Moreover, the intensity of cyclical mastalgia was determined by visual analogue pain intensity scale. In case any participant could not make the visit before their menstrual periods, the visit was arranged for 2 or 3 days after the beginning of the periods. To follow up the effectiveness of the intervention in cyclical mastalgia, in the third cycle when the intervention was stopped, the participants were visited and the intensity of their cyclical mastalgia was redetermined by the visual analogue scale.

### 2.3. Sample Size and Statistical Analysis

By taking the study objectives and the results of other similar studies into account, considering the error rate of 5%, power of 90%, 2.2 maximum combined standard deviation, 1.3 effect size, and the number of study groups (*k* = 3), and using the following formula and the SPSS software, a 180-subject sample size (60 in each group) was determined for the study:

(1) $$\begin{array}{c} n=\frac{2\phi^{2}{Ks}^{2}}{{\left(\partial \right)}^{2}}. \end{array}$$

The data were analyzed using the SPSS statistical software (ver. 16). Besides, *P* values of less than 0.05 were considered as statistically significant. Considering the relatively large sample size of the study and utilization of visual analogue scale for determining mastalgia intensity which is numerated from 0 to 10, parametric tests were used for data analysis. Therefore, in order to compare pain intensity among the three groups in different stages (baseline, first and second interventional cycles, and the third nonintervention cycle), one-way analysis of variance and analysis of covariance were used. In addition, repeated measures analysis of covariance was applied for intragroup comparison of mastalgia intensity in various stages. Chi-square and *t*-test were also used for parametric variables.

## 3. Results

**Figure 1:**
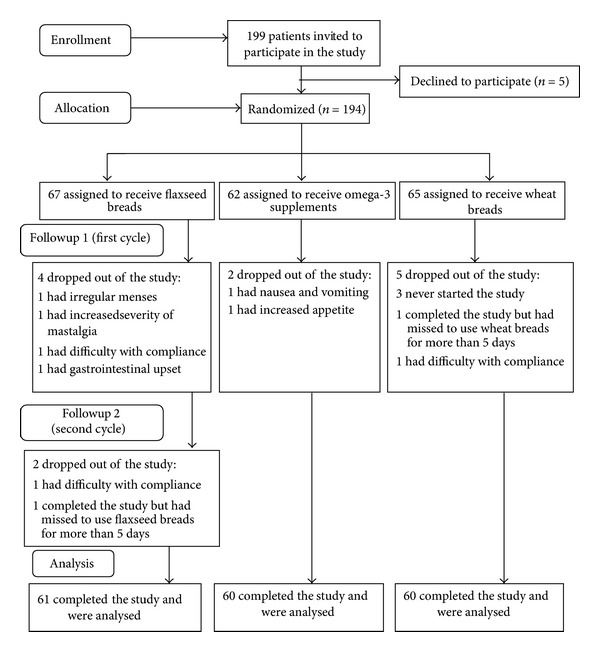
The flow of clinical trial.

A total of 199 women with mastalgia were interviewed to enter the study. The excluded cases along with their reasons have been shown in the Figure 1. After all, 181 women with cyclical mastalgia finished the two interventional cycles. Out of these 181 women, 61, 60, and 60 were located in flaxseed, omega-3, and wheat bread intervention groups, respectively. The mean age of the study participants was 29.63 ± 7.05 years. Besides, the mean age of the participants in flaxseed, omega-3, and wheat bread groups was 32.07 ± 6.7, 29.58 ± 8.01, and 27.3 ± 5.64 years, respectively, and the difference was statistically significant (*P* = 0.001). Most of the participating women (99 participants, 54.7%) were homemakers. Additionally, 49.7% of the subjects (90 participants) were single and 42% (76 participants) held a high school diploma. The mean of menstrual intervals was 28.1 ± 2.56 days and a slight difference was observed among the three groups in this regard (*P* = 0.049). Most of the participants (151, 83.4%) had bilateral cyclical mastalgia. In addition, 13.8% (25 participants) had unilateral cyclical mastalgia and 2.8% (5 participants) sometimes had bilateral and sometimes suffered from unilateral mastalgia. In this regard, no significant difference was observed among the three groups.

The mean intensity of cyclical mastalgia was 6.14 ± 1.9 in the baseline cycle. Comparison of the intensity of mastalgia using analysis of variance showed a statistically significant difference among the three groups. In order to compare mastalgia intensity in the first, second, and third cycles, analysis of covariance was applied. The results which have been presented in Table 1 showed a significant difference among the three groups in all the three cycles. As mentioned above, a significant difference was found among the three groups regarding their mean age and mean baseline mastalgia intensity. Therefore, repeated measures analysis of covariance was used to adjust these two parameters (Table 2). According to the results, the intensity of mastalgia was significantly lower in the flaxseed diet group compared to omega-3 and wheat bread groups (*P* < 0.001). Furthermore, the intensity of mastalgia was lower in the omega-3 intervention group in comparison to the wheat bread intervention group (*P* = 0.003).

Intragroup analysis showed that the intervention in two menstrual cycles reduced the mean intensity of cyclical mastalgia compared to the baseline. Also, the mean intensity of cyclical mastalgia was decreased in the third cycle (no intervention) in comparison to basic mastalgia intensity. Repeated measures analysis of covariance indicated a significant difference among the four menstrual cycles (baseline, first, second, and third cycles) concerning mastalgia intensity (Table 1). In the flaxseed bread group, the intensity of mastalgia in the first (*P* = 0.02), second, and third (*P* < 0.001) cycles was statistically different from that at the baseline cycle. However, in the wheat bread group, only a significant difference was found between mastalgia intensity in the second interventional cycle and the baseline one (*P* = 0.01). In the omega-3 group also, the intensity of mastalgia intensity in the second (*P* < 0.001) and third (*P* = 0.06) interventional cycles was different from that at baseline.

In this study, the mean duration of mastalgia was 5.85 ± 1.52 days, which decreased to 3.95 days in the second interventional cycle. The longest duration of mastalgia was 14 days. The results showed no significant difference among the three groups regarding the duration of mastalgia in the baseline cycle (*P* = 0.37). In the second interventional cycle, however, a significant difference was observed in the three groups regarding the mean mastalgia duration (*P* = 0.009). After the two interventional cycles, flaxseed bread, wheat bread, and omega-3 groups, respectively, showed 23% (14 subjects), 16.7% (10 subjects), and 13.3% (8 subjects) change in the duration of their menstrual cycles, but the difference was not statistically significant (*P* = 0.37).

## 4. Discussion

In the present study, one group of women with cyclical mastalgia received 30 g flaxseed mixed with wheat flour added to their normal diet for two menstrual cycles. The other two study groups received wheat bread slices or pearls of omega-3 fatty acids. The results showed that flaxseed bread diet was more effective in decreasing mastalgia intensity in comparison to wheat bread and omega-3 fatty acids. Besides, omega-3 was more effective than wheat bread. The only study on the effect of flaxseed on cyclical mastalgia is that of Goss, which was in good agreement with the present study [20]. Also, the study by Ingram et al. showed that 40 and 80 mg isoflavones, the main phytoestrogen existing in soy, were effective in reducing cyclical mastalgia [28].

Up to now, no studies have focused on comparison of the effects of flaxseed and omega-3 fatty acids on cyclical mastalgia. However, Sohrabi et al. investigated the effect of omega-3 fatty acids on premenstrual syndrome and reported that omega-3 fatty acids decreased breast tenderness and affected some other symptoms [23]. The flaxseed used in this study was ground whole flaxseed which contained a considerable amount of omega-3 fatty acids in addition to phytoestrogens. The superiority of flaxseed to pearls of omega-3 in reducing the intensity of mastalgia might be due to the interaction of these two elements. Furthermore, the lignan available in flaxseed might have a better effect compared to omega-3 fatty acids found in pearls. In the current study, more than 80% of the participants suffered from bilateral cyclical mastalgia, while most of the participants in Cheung's study in Hong Kong had unilateral left mastalgia despite being right-handed [29].

According to the literature, cyclic mastalgia is more frequent in the third and fourth decades of life [2, 4]. In this study, the mean age of the participants was 29.63 ± 7.05 years, which is consistent with the literature. It should be noted that age could have an effect on the outcomes of the interventions; thus, the parameter of age was adjusted. Afterwards, it was observed that age did not affect the severity of mastalgia after the interventions.

In this study, consumption of flaxseed was accompanied by some side effects, such as flatulence and diarrhea, which were similar to those of other studies [13]. After the interventions, no significant difference was observed among the three groups regarding the change in menstrual intervals. Thus, short-term consumption of 30 g lignan-containing flaxseed did not affect the menstrual intervals and could be safe in this regard.

At the beginning of the study, all the participants had clinical breast examination and were assured that their mastalgia was not related to breast cancer. Some studies have reported that such an assurance could decrease the intensity of cyclical mastalgia in women [30]. Yet, this decrease is mostly observable in mild mastalgia cases, while the participants of the current study suffered from moderate to severe mastalgia.

The intervention period was relatively short in our study (two menstrual cycles). Although the mean intensity of mastalgia increased in the third cycle compared to the second one, lower mastalgia intensity in the third cycle compared to the baseline can demonstrate the effectiveness of the interventions. Nevertheless, further studies with longer duration of interventions should be conducted in order to investigate the continuation of the effectiveness of flaxseed and omega-3 fatty acids.

In this study, the amount of blood lignan or fatty acids was not measured in the study groups and the work was based on the participants' self-reports about consuming the pearls or bread. Therefore, further studies are recommended to use laboratory information for investigating the effect of flaxseed and omega-3 fatty acids on cyclical mastalgia.

## 5. Conclusions

The findings of this study demonstrated that flaxseed bread was effective in decreasing the intensity of cyclical mastalgia and could be considered as a simple method with few complications for women. Furthermore, omega-3 fatty acids could be regarded as an alternative for treating cyclical mastalgia. We hope these results to be confirmed in future studies.

## Figures and Tables

**Table 1:** Comparison of the means of mastalgia intensity: between-group analysis, within-group analysis.

	Flaxseed group (Mean ± SD)	Wheat group (Mean ± SD)	Omega-3 group (Mean ± SD)	*P* value (analysis of variance)	*P*-value (analysis of covariance)
Baseline cycle	6.49 ± 1.17	5.85 ± 1.03	6.08 ± 1.07	0.009	

First interventional cycle	3.66 ± 2.51	4.92 ± 2.06	4.43 ± 2.34	0.008	<0.001

Second interventional cycle	2.89 ± 2.46	4.85 ± 1.99	3.53 ± 2.13	0.001	<0.001

Third cycle (stop the intervention)	2.94 ± 2.29	4.95 ± 1.68	2.94 ± 2.29	0.001	<0.001

*P* value	<0.001	<0.001	<0.001		

**Table 2:** Comparison of the means of mastalgia intensity after adjusting the age and mastalgia intensity at the baseline cycle.

Variable	Mean	Standard deviation	*P* value
Age	29.63	7.05	0.979
Mastalgia intensity in the baseline cycle	6.14	1.12	<0.001
Group			
Flaxseed group	2.76	1.62	<0.001
Wheat group	4.99	1.62	
Omega-3 group	3.99	1.56	
